# MYC and Metabolomics: Can We Use What We Know for DLBCL Subtyping and Diagnosis?

**DOI:** 10.3390/biom15091346

**Published:** 2025-09-20

**Authors:** Adrian Florentin Suman, Davide De Luca, Melania Gaggini, Francesco Cucco

**Affiliations:** Institute of Clinical Physiology, National Research Council, 56124 Pisa, Italy; adrianflorentinsuman@cnr.it (A.F.S.); davidedeluca@cnr.it (D.D.L.)

**Keywords:** amino acids, metabolomics, biomarkers, lymphoma, MYC, diagnosis

## Abstract

Diffuse large B-cell lymphoma (DLBCL) is a molecular and clinical heterogenous entity, and, over the past 30 years, many efforts have been made in trying to dissect this diverseness and identify biomarkers capable of efficiently stratifying DLBCL patients and spotting the ones showing a worse clinical outcome. Despite the achievement in this research field, only a few biomarkers have been validated and introduced in a clinical setting. Among those, approximately 5–15% of DLBCL cases harbor *MYC* gene translocations, often involving immunoglobulin genes as a translocation partner, and concomitant point mutations, correlating with a poor response to standard therapies. However, given the difficulty in detecting these abnormalities requiring specialized techniques and high-quality specimens, the use of metabolomics (i.e., the study of small metabolites in body fluids and tissues) can offer a useful alternative for the identification of high-risk DLBCL patients. Amino acids (AAs) are metabolites essential in the process of tumorigenesis and can increase immune escape and drug resistance. Therefore, we review the use of metabolomics to improve the diagnosis and prognosis in DLBCL patients in relation to the MYC role in the regulation of amino acid metabolism, as these metabolites may be used as potential biomarkers in a clinical environment.

## 1. Introduction

DLBCL is a group of nodal and extranodal large B-cell lymphomas that account for about 30% of adult lymphomas and include a heterogeneous variety of clinically and molecularly different cases [1,2]. The standard therapy is R-CHOP (rituximab plus cyclophosphamide, doxorubicin, vincristine, and prednisone) chemoimmunotherapy, which achieves a five-year survival rate in about 70% of patients [2]. The proportion of patients relapsing after R-CHOP poorly respond to other therapies. How to predict patients who do not respond to therapies and treat them according to their molecular architecture is a huge unmet clinical need. DLBCL carrying rearrangements of *MYC* and B-cell lymphoma 2 (*BCL2*) genes and/or B-cell lymphoma 6 (*BCL6*), detected by fluorescence in situ hybridization (FISH), were introduced in the 2017 World Health Organization (WHO) classification update as defining alterations of a novel category named “High Grade B-cell lymphoma with *MYC*, *BCL2* and/or *BCL6* rearrangements”, known to poorly respond to R-CHOP. This category was maintained by the most recent classifications [1,2] including exclusively high-grade B-cell lymphoma (HGBL) with *MYC* and *BCL2* rearrangements (*MYC*/*BCL2*). Furthermore, when the *MYC* rearrangement partner is represented by the immunoglobulin genes (*IG*), double hit lymphomas (*MYC*/*BCL2*-DH) are characterized by a worse clinical outcome, but the reason for this is unclear [3]. This may be explained, at least partially, by the finding of frequent *MYC* point mutations in DLBCL cases with a *MYC*/*IG* gene translocation (~65%), compared with cases harboring a *MYC*/non-*IG* rearrangement (~13%) [4]. The *MYC* mutation profile, in those cases, is consistent with the one found in Burkitt Lymphoma (BL), where almost all the cases are characterized by the same translocation [5]. Among the DLBCL *MYC* point mutations, there is a clear hotspot affecting the MYC phosphorylation site (T58) and its adjacent residues [4]. These hotspot mutations are critical for F-Box and WD Repeat Domain-Containing 7 (FBXW7)-mediated proteasome degradation and have been proven to increase the MYC protein half-life in vitro [6]. In addition, these mutations have been found to significantly co-occur with *MYC*/*IG* genes translocations, correlating with unfavorable clinical outcomes and higher levels of MYC protein expression by immunohistochemistry (IHC) [4]. This evidence suggests that MYC upregulation is a driver event in lymphoma development and maintenance, and this is often linked to the pro-proliferative phenotype typically induced by MYC aberrant expression/activity [7]. However, MYC is a key factor in regulating multiple aspects of cell biology including cell cycle progression, apoptosis, DNA replication, protein synthesis, and metabolism [8,9]. Recently, it has been demonstrated that there is a harmful interaction between MYC inhibition and mitochondrial metabolism, since the disruption of mitochondrial complex I creates a unique vulnerability in MYC-inhibited cells [10]. Furthermore, important interactions with MYC and Hypoxia-Inducible Factor-1 alpha (HIF-1α) have been observed, which promote glucose and glutamine metabolism and the activation of antigen presentation on regulatory T cells and their subsequent metabolic reprogramming [11]. Moreover, MYC is involved in the regulation of the AAs metabolism, which correlates with tumor development and progression, since cancer cells have a consistent demand for AAs to provide substrates for energy production [8,12]. MYC activates critical transporters for essential amino acids, including branched amino acids (BCAAs), in particular, solute carriers (SLC) *LAT1*, *LAT3*, and Solute Carrier Family 1 Member 5 (*SLC1A5*). MYC also targets branched-chain amino acid transaminase 1 (*BCAT1*), which catalyzes the decomposition of BCAAs [13]. In MYC-driven B-cell lymphomas, MYC enhances the translation of glutaminase (*GLS*), which converts glutamine (Gln) into glutamate, which is then further metabolized in the TCA cycle, providing intermediates for energy synthesis (ATP), a fundamental process for the high proliferation rate of these cancerous cells [14]. These and other aspects of the MYC biology in lymphomas with a focus on DLBCL and AAs metabolism will be discussed more in depth in the subsequent chapters of this review. 

## 2. DLBCL Molecular Subtyping

DLBCL is a heterogenous entity, and many efforts have been made by researchers in the past 30 years to dissect this complexity, particularly at the molecular level. 

**Figure 1:**
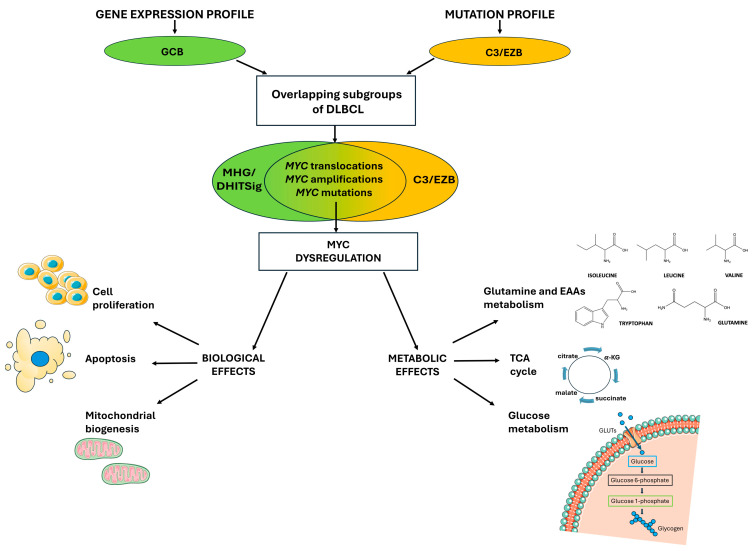
Similar *MYC* gene alterations leading to MYC deregulation were found by two different classification systems in overlapping subsets of DLBCL cases. Two independent molecular classification systems, gene expression profiles, and mutational profiles were identified in DLBCL, the MHG/DHITSig group, and the EZB-MYC+ group, respectively. These subsets of patients share *MYC* locus alterations, e.g., chromosomal rearrangement, copy number gain, and point mutations, which lead to MYC dysregulation; indeed, these genetic abnormalities promote MYC overexpression or enhance its constitutive activity, with dramatic impact both on cellular proliferation and metabolic reprogramming.

The first successful attempt was a classification based on the gene expression profile (GEP), which identified DLBCL cases sharing similar expression signatures with two specific stages of B-cell maturation: germinal center B-cell (GCB) and activated B-cell (ABC) [15]. This early classification identified the Cell-Of-Origin (COO) of these lymphomas and showed promising results in the clinic, with the ABC subtype displaying a worse outcome within an R-CHOP regimen [16]. More recently, advances in the DLBCL GEP-based classification allowed the refinement of this kind of molecular subtyping up to the identification of the so-called Molecular High-Grade (MHG) subtype, which represented a further step in the stratification of the GCB group [17] (Figure 1). Indeed, the MHG has a gene expression signature like the centroblast in the dark zone of the germinal center (GC) and distinguishable from one of the centrocytes of the GC light zone [17]. Moreover, the MHG signature was originally designed to distinguish DLBCL from BL, which is a (mostly) pediatric and aggressive type of lymphoma [18]. Consistently, the MHG group in the clinic showed a worse prognosis among the GEP subtypes, and this discovery was particularly relevant as the MHG largely belonged to the GCB group, which globally showed a favorable outcome [17]. The same results have been achieved independently by another group by using a slightly different GEP approach, defining the Double-Hit Gene Expression Signature (DHITSig), since cases with this signature were like HGBL-*MYC*/*BCL2* rather than BL and centroblasts [19]. However, about half of the cases with MHG or DHITSig do not present *MYC* and *BCL2* rearrangements [17,19], indicating that additional mechanisms are likely involved and can be caught by the application of specific gene expression signatures.

DLBCL GEP-subtypes also showed distinct mutational profiles [20], and this raised the possibility of using genetic alterations for the identification of well-defined groups. To this aim, two independent studies used a whole exome sequencing (WES) approach applied to large cohorts of DLBCL cases and defined a few genetic categories which largely overlapped [21,22]. Further refinement of this classification identified the EZH2-mutated and *BCL2*-translocated (EZB)-MYC+ group, which was characterized by a BL-like mutation profile and ribosomal protein expression signature, therefore sharing molecular features with the MHG/DHITSig GEP group [23] (Figure 1). Altogether, this evidence suggests that DLBCL molecular profiling could potentially be used in the clinic for precise diagnosis and treatment decisions. However, to date, these analyses are not easily appliable in routine clinical practice, and more simplified markers are required.

## 3. MYC-Driven Lymphomas

Genetic rearrangements involving *MYC* locus are a predominant mechanism leading to *MYC* overexpression in multiple hematological cancers [24]. *MYC* translocations were firstly observed in BL, where 90–95% of these alterations are represented by the t(8;14) (q24;q32) rearrangement. This translocation normally puts *MYC* under the control of the Ig heavy-chain (*IgH*) gene’s strong regulatory elements, causing uncontrolled *MYC* expression. The remaining 5–10% of BL cases present t(2;8) (p12;q24) and t(8;22) (q24;q11) rearrangements [25,26]. *MYC* translocations are not only observed in BL, but also in other B-cell malignancies. Indeed, in DLBCL, *MYC* translocations are found in 5–15% of patients, rising to 50% when considering DLBCL cases which also present *BCL2* and/or *BCL6* rearrangements [27]. Moreover, a EUROMYC retrospective study found a high frequency of *MYC* translocations in HIV-associated large B-cell lymphomas, consistent with their aggressive nature, and suggesting the necessity of differential and more intensive therapies [28].

Aside from chromosomal rearrangements, MYC deregulation in lymphomas is also caused by other genetic alterations such as *MYC* copy number gains. Zak and colleagues investigated the prognostic impact of *MYC* amplifications in a cohort of 396 large B-cell lymphoma (LBCL) patients. The authors observed that 20.9% of cases presented *MYC* amplification, with a correlation between a high copy number gain (>7 *MYC* copies) and worse prognosis [29]. Comprehensively, *MYC* chromosomal translocations and *MYC* amplifications are also found in other aggressive hematological neoplasms such as plasmablastic lymphoma, transformed follicular lymphoma, and lymphoblastic leukemia [27,28].

Even if *MYC* dysregulation in lymphomas is mostly associated with chromosomal translocations and, to a lesser extent, copy number gains, *MYC* point mutations represent another type of *MYC* alteration, which have been firstly observed in BL [30]. *MYC* point mutations have been traditionally considered a minor oncogenic factor with respect to *MYC* amplifications and chromosomal rearrangements due to their relative low frequency. However, in the last 20 years, the routine use of next-generation sequencing approaches allowed for the identification of *MYC* point mutations in various cancer types, including both solid and non-solid tumors, often in association with poor outcomes [31,32,33]. The origin of these mutations in lymphomas is uncertain, but *MYC* is a predicted target of the aberrant somatic hypermutation machinery (aSHM), which displays a high affinity for the genomic regions around the transcription start site (TSS) of transcriptionally active genes. Indeed, the distribution of *MYC* point mutations in lymphomas recapitulate an aSHM mutation-driven signature [34]. Despite these considerations, the functional consequences of *MYC* point mutations are still mostly uncharacterized and need further investigation, although there are some exceptions such as the cluster of hotspot mutations affecting the Myc Box I (MBI) domain. Multiple experimental evidence showed that MBI point mutations are critical for MYC turnover and function; indeed, these mutations have been shown to affect the FBXW7-mediated proteasome degradation, increasing the MYC protein half-life in vitro [35,36,37].

In general, B-cell lymphomas driven by MYC dysregulation are characterized by genomic instability and metabolic reprogramming to support rapid cell proliferation. *MYC* alterations in hematological cancers differ from the ones observed in solid tumors, e.g., the former predominantly present *MYC* translocations and point mutations, while the latter are mostly characterized by *MYC* amplifications [24].

## 4. MYC Dysregulation: From Gene Expression to Metabolic Reprogramming

The *MYC* gene encodes for the MYC proto-oncogene, a transcription factor that regulates the expression of 10–15% of the genome [38]. At the protein structure level, it is organized in an N-terminal transactivation domain and a C-terminal basic helix-loop-helix–leucine zipper (bHLH-LZ) domain. In particular, the bHLH-LZ is crucial for the heterodimerization with partner proteins, such as MYC Associated Factor X (MAX), for transcription activation [39]. MYC-MAX complexes bind consensus DNA sequences, the E-boxes (5′-CACGTG-3′) domains in the promoter of target genes involved in many cellular processes, including cell metabolism, cell growth, and survival, to regulate their expression [39].

Within healthy cells, MYC expression is controlled both at the transcriptional and post-transcriptional level to finely tune, respectively, the production of its messenger RNA (mRNA) and its translation rate. These control mechanisms are essential, because even small perturbations in MYC expression and activity display a high oncogenic capacity [40]. Indeed, MYC promotes the activation of genes involved in the G1–S phase transition within the cell cycle, whereas it represses the activity of cell cycle inhibitors such as Cyclin-Dependent Kinase Inhibitor 2B (CDKN2B). Therefore, MYC overexpression results in the reinforcement of these mechanisms, ultimately leading to an increased cell proliferation rate [41]. On the other hand, normal cells react to MYC hyperactivity by triggering apoptosis; in fact, uncontrolled and sustained MYC expression can lead to proliferative stress and the subsequent activation of apoptotic processes, such as the Alternate Reading Frame (ARF)–mouse double minute 2 homolog (MDM2)–Tumor Protein p53 (TP53) axis [41]. Therefore, additional genetic alterations are necessary to allow MYC to exert its oncogenic activity in cancer cells, e.g., the enhanced expression of antiapoptotic factors such as *BCL2* (through chromosomal translocations) or mutations in checkpoint genes like *TP53* [42] (Figure 1).

In addition, MYC regulates mitochondrial biogenesis by the expression of nuclear-encoded mitochondrial genes involved in mitochondrial mass regulation. Among these genes, the most relevant are the mitochondrial transcription factor A (*TFAM*), the peroxisome proliferator-activated receptor gamma coactivator 1-alpha (*PPARGC1A*), and several members of the Translocase of the Outer Mitochondrial Membrane (TOM) and Translocase of the Inner Mitochondrial Membrane (TIM) import machinery [43]. In particular, the TIM/TOM machinery is a sophisticated system for recognition and translocation across the outer and inner mitochondrial membranes of almost 1000 precursors of proteins involved in mitochondrial structure and function [43] (Figure 1).

Exploring the MYC-regulated metabolic activity within cells, MYC controls nutrient acquisition to produce ATP and key cellular metabolites to increase cell mass and trigger DNA replication and cell division programs by regulating glutaminolysis, nucleotide synthesis, and glycolysis [43]. Indeed, glycolytic enzymes, e.g., Glucose Transporter 1 (*GLUT1*), Hexokinase 2 (*HK2*), Phosphofructokinase (*PFKM*), Enolase 1 (*ENO1*), and Lactate Dehydrogenase A (*LDHA*), are upregulated by MYC, which also promotes alternative splicing of the pyruvate kinase-encoding gene, favoring the Pyruvate Kinase M2 (*PKM2*) isoform [44]. Therefore, MYC enhances the uptake of glucose with the consequent production of lactate to generate rapid high levels of energy, i.e., the Warburg effect [44]. Furthermore, MYC upregulates *GLS* and the AAs transporter, e.g., Solute Carrier Family 1 Member 5 (*SLC1A5*), to increase the glutamine uptake and, by supporting glutamine anaplerosis, MYC contributes to the replenishment of intermediates of the tricarboxylic acid (TCA) cycle [45]. Given MYC’s role in the metabolic regulation of nutrient uptake to meet the high energy requirements for tumor proliferation, specific metabolites regulated by MYC are currently studied through metabolomic techniques to identify potential diagnostic and prognostic markers in solid and non-solid tumors. In the next chapter we will review the use of these applications in B-cell lymphomas with a particular focus on DLBCL.

## 5. The Use of Metabolomic Techniques in B-Cell Lymphoma for Diagnosis and Prognosis

Cancer cells have a dysregulated metabolism due to uncontrolled proliferation. The most obvious metabolic modification in tumors is the increase in glucose consumption, which results in more lactate production, even when oxygen is present, as was widely demonstrated many years ago [46,47]. However, tumor cells have been shown to support cancer growth, survival, and invasion by increasing the uptake of other nutrients (such as Gln and fatty acids) [48]. The study of metabolic alterations through metabolomics is a powerful tool that can identify cancer metabolites and drivers of tumorigenesis. Metabolomics is based on high specificity and sensibility techniques that can detect and quantify a wide spectrum of metabolites [49]. Metabolomics studies can be divided into two classes: targeted and untargeted. The targeted workflow focuses on the quantitative measurement of predefined groups of known metabolites, often driven by specific biological hypotheses. Untargeted metabolomics aims to detect as many metabolites as possible in a sample, including unknown compounds, without a prior hypothesis [50]. For the metabolomic analysis of biological fluids, there are different techniques, and the platform of choice depends on experimental goals, sample traits, and sensitivity. The most analytical platforms used are Gas Chromatography–Mass Spectrometry (GC-MS), Liquid Chromatography–Mass Spectrometry (LC-MS), and nuclear magnetic resonance (NMR) spectroscopy (Table 1) [51,52].

Metabolites play a role as signaling molecules, energy precursors, and cofactors, and they also regulate various processes and enzymatic cascades [59,60,61]. Additionally, metabolites, particularly AAs, are involved in the biological processes of tumorigenesis, cancer cell invasion, cancer stem cell pluripotency, insulin sensitivity, epigenetic regulation, and other cellular processes [62]. To date, there are few studies that concern metabolomic analysis and AA profiles in DLBCL. Regarding the mass spectrometry techniques applied to metabolomics, we report below some works on the evaluation of AAs related to diagnosis or prognosis in DLBCL. The use of the GC-MS technique has been used in some studies on subjects with DLBCL in different types of matrices (i.e., plasma, urine). Barberini et al. used untargeted GC-MS to compare the metabolic profile by case/control status and across the major lymphoma subtypes. They showed that glycine was more abundant in the plasma samples of DLBCL compared to the controls [53]. In another study, by the use of untargeted GC-MS, the urine samples of patients with DLBCL and healthy individuals were investigated, and the results showed that glycine, serine, threonine, and their metabolisms, as well as aminoacyl-tRNA biosynthesis, were the metabolites with the highest capacity to discriminate healthy subjects from DLBCL patients. Therefore, these metabolites could be exploited as potential diagnostic biomarkers and targets for therapeutic purposes. [54]. Moreover, an untargeted plasma metabolomics analysis by the GC-MS technique was carried out in the healthy controls (Ctrl), DLBCL patients that were recently diagnosed (ND), and DLBCL patients who achieved complete remission (CR) as part of another study. The aim was to explore the metabolic characteristics of peripheral plasma derived from DLBCL patients and the Ctrl and identify metabolic pathways and biomarkers associated with the diagnosis and prognosis prediction of DLBCL. The results showed that the level of glucose and aspartate in plasma was decreased in ND patients and elevated in CR patients, indicating that glucose and aspartate may be involved in the pathogenesis of DLBCL [55]. The lower level of aspartate in ND patients may be related to disorders of the asparagine metabolism. Furthermore, a decrease in methionine and cysteine as well as in branched-chain amino acids was observed in the plasma of ND patients, and this alteration was closely associated with a worse prognosis [55]. The GC-MS technique was employed in another study for the detection of metabolic alterations, which could be specifically assigned to the DLBCL Cell-Of-Origin subtypes, and with the aim to identify some metabolites associated with the clinical outcomes. Unfortunately, metabolomics profiling did not distinguish the GCB subtype from the non-GCB subtypes; nevertheless, serum metabolomic profiling reported that valine and pyroglutamic acid could be candidate biomarkers for the prognosis of DLBCL [56]. LC-MS was used to perform untargeted metabolomic studies on serum samples from patients with newly diagnosed DLBCL and whose age–gender matched healthy controls to verify whether the differential metabolites had diagnostic value in DLBCL patients. The metabolic profile of DLBCL patients was distinct from the healthy controls, indicating a differential metabolism in the disease condition. The enrichment analyses showed that differential metabolites were mainly enriched in the D-Glutamine and D-glutamate metabolism. In particular, the Gln and glutamate metabolism are essential for cell survival and proliferation, since DLBCL cells are largely dependent on the concentration of Gln, which is converted to glutamate by GLS [50]. Glutamate, in turn, is used for various purposes: it provides nitrogen for nucleotide and AA synthesis, generates alpha-ketoglutarate (α-KG) for the TCA cycle, and even acts as a signaling molecule [57]. The use of NMR-based serum metabolomics can help to identify a signature for patients with DLBCL who are at high risk of failing immunochemotherapy, leading to more personalized treatment. The serum metabolomic signature at the time of diagnosis, analyzed by NMR spectroscopy, seems to differ between DLBCL patients with refractory disease or early relapse (REF/REL) and cured (CURED) patients. Some AAs (lysine and arginine) were significantly higher in REF/REL patients. In contrast, the AAs aspartate, valine, and ornithine were found to be higher in the cured patient group [58].

## 6. MYC-Driven Amino Acid Metabolism in Cancer Cells

**Figure 2:**
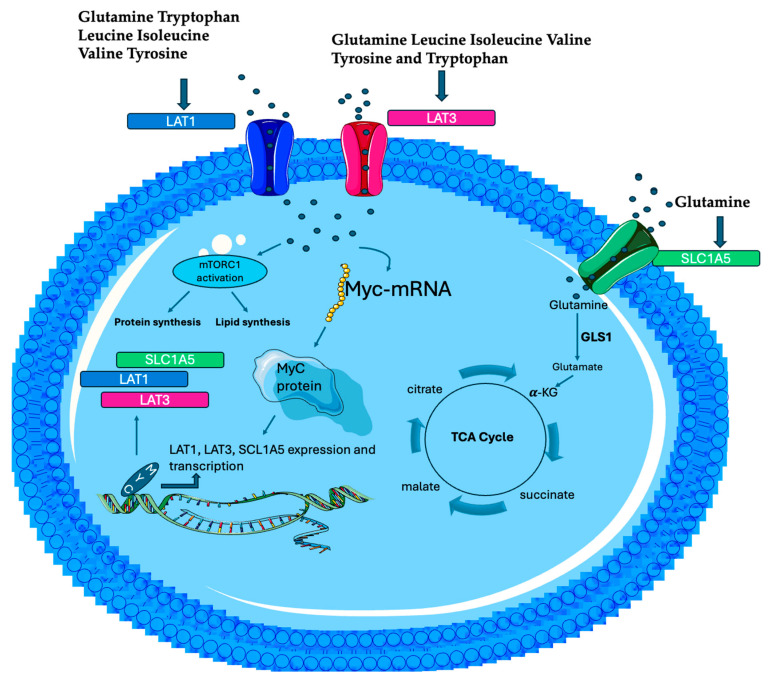
MYC selectively activates LAT1, LAT3, and SLC1A5 expression and transcription. LAT1 and LAT 3 import into the cell EAAs, more specifically, BCAAs (leucine, valine, and isoleucine), tryptophan, as well as glutamine. The increase in EAAs, in turn, stimulates MYC mRNA translation, leading to more MYC protein, constituting a feedforward activation loop to promote the EAAs’ transport and tumorigenesis. MYC promotes Gln uptake by activating the expression of glutamine transporter *SLC1A5* and GLS1 or GL2 convert Gln into glutamate, that is then further metabolized in the TCA cycle, providing intermediates for energy (ATP). Moreover, EAAs activate mTORC1 pathways that promote protein and lipid synthesis, crucial processes for growth and repair processes.

Animal cells frequently exhibit an increased demand for essential amino acids (EAAs), because cells cannot synthesize EAAs de novo or in insufficient amounts, requiring these from diet [63]. AAs, as carbon and nitrogen donors, allow to get rid of the nutrition limitation and therefore play an important role in tumorigenesis. AAs have a regulatory function in tumors, participating in signaling pathways, the tumor microenvironment, and epigenetic modifications [64]. AAs metabolism, both EAAs and non-essential amino acids (NEAAs), are regulated by MYC, which also modulates metabolic reprogramming, cancer cell anabolism, metabolic enzymes expression, mitochondrial respiration, glycolysis, and fatty acid and nucleotide synthesis in different types of tumors [65]. In cancer cells, MYC plays a substantial role in promoting the transcription of the L-type amino acid transporter (*LAT*) (Na+-independent transporters), which transports neutral AAs into cells. LAT expression has been found to be increased in many cancers, and it is essential for cell growth and protein translation regulation through the mTORC1 pathway [66,67]. AAs are essential for the activation of mTORC1, which in turn increases lipid and protein synthesis [68]. The transport of neutral AAs into cells is mediated by a membrane transporter family, grouped into two sub-families, specifically SLC7 (LAT1 and LAT2) and SLC43 (LAT3 and LAT4). MYC selectively upregulates LAT1 and LAT3 expression and transcription [67,69]. In Burkitt’s lymphomas, oncogenic MYC and LAT1/LAT3 transporters create positive feedback called MYC-LAT1/LAT3. MYC activates *LAT1* and *LAT3* expression and promotes effective EAAs uptake into tumor cells (Figure 2) [70]. Efficient EAAs incorporation, in turn, promotes MYC synthesis, constituting a feedforward activation loop that reinforces MYC-regulated oncogenic programs, conferring advantages to acquiring nutrients and utilizing nutrients during malignant transformation. Consistently, in MYC-overexpressing tumor cells, LAT1/LAT3 inhibition compromises metabolic reprogramming and, in particular, the depletion of LAT1 significantly inhibits glucose and Gln uptake [69]. MYC can also regulate NEAAs metabolism, in particular, the AAs Gln, proline, and serine. In cancer cells, Gln is the most abundant AA in blood circulation, acting as major fuel, and anaplerosis metabolites in tumors, driving the TCA cycle to sustain mitochondrial ATP production. Anaplerotic metabolism of Gln generates α-KG and subsequently oxaloacetate (OAA), fueling the TCA cycle through a series of biochemical reactions termed glutaminolysis [71]. Gln maintains redox homeostasis in numerous metabolic processes and is essential for the proliferation of many cancer cells, regulating mitochondrial functions and macromolecular synthesis [72]. MYC promotes Gln uptake by activating the expression of glutamine transporter *SLC1A5* [69,73]. Glutaminolysis is a process catalyzed by GLS1 or GLS2 to produce glutamate and fuel the TCA cycle *(*Figure 2)**.** GLS1 and GLS2 are isozymes that play opposite roles in tumor development. GLS1 has oncogenic properties, while GLS2 has been described as a tumor suppressor [74]. In human P-493 B lymphoma cells, MYC transcriptionally represses miR-23a/b to enhance the translation of *GLS1*, leading to an increase in glutaminolysis. P-493 B-cell growth was diminished significantly by Gln withdrawal, that also led to the decrease in ATP levels, reflecting diminished cellular oxygen consumption [14]. Cancer cells need free AAs because of their highly anabolic state; for this reason, the autophagy–lysosome circuit is upregulated in cancer cells [75], and Transcription Factor EB (TFEB), a master regulator of the autophagy–lysosome pathway, sustains AA pools via protein breakdown. In acute myeloid leukemia, MYC suppresses autophagy by antagonizing the expression and function of TFEB, and this suppression circuit is necessary for the maintenance of a malignant state [76,77]. In normal B cells and in premalignant and neoplastic B cells of Em-Myc transgenic mice, the autophagy pathway is suppressed by MYC. In this regard, given the suppression of autophagy driven by MYC, it was evaluated whether there were changes in intracellular AA pools in normal versus premalignant Em-Myc pre-B cells. Concentrations of AAs were significantly increased (BCAAs, Gln, glutamate, glycine, and arginine) in premalignant Em-Myc pre-B cells versus normal pre-B cells, indicating that MYC-expressing B cells have compensatory mechanisms to maintain AA pools [70]. In view of all these considerations, altered AA metabolisms are common in tumors, particularly lymphomas; thus, targeting molecules in AAs’ metabolic signaling pathways has the potential to treat cancer.

## 7. Conclusions

Metabolism alteration in cancer cells is one of the most important aspects in the discrimination between healthy and cancerous cells. As mentioned, AAs contribute to tumorigenesis, acting as a source of energy for tumor growth, driving alterations in the cellular metabolism to meet the increased demand for nutrients [76]. Targeting AA metabolisms, trying to inhibit the transporters and enzymes that regulate the supply of AAs, could be an important approach in cancer therapy. Gln is the most consumed amino acid, and its availability can limit tumor cell proliferation; it is utilized for the biosynthesis of NEAAs, and is also the major source of α-ketoglutarate in the TCA cycle, besides the fact that its intermediate glutamate functions as an exchange factor for the import of EAAs [78]. Tumors driven by MYC or KRAS are particularly dependent on exogenous Gln. Glutaminolysis and the de novo biosynthesis of Gln are upregulated in several cancers via common oncogenes/tumor suppressors including MYC and p53 [79]. Gln synthesis is often due to the upregulation of Gln synthetase, whereas enhanced glutaminolysis is caused by increased GLS activity [80]. Various inhibitors of GLS have been proposed to counteract glutamine catabolism, in particular CB-389, a potent, selective, and orally bioavailable inhibitor of both splice variants of glutaminase, which showed significant antitumor activity in two xenograft models [81]. Moreover, MYC upregulates LAT1 and LAT3 expression and transcription, sustaining EAA metabolism in tumor cells. The therapeutic approach aimed at inhibiting LAT1 would offer an opportunity to unleash the functional association between MYC and LAT1, leading to tumor suppression. JPH203, a specific LAT1 inhibitor, can play a role as a novel anti-tumor agent and can be evaluated as an MYC-selective cancer therapeutic in future clinical trials [82].

The metabolomic approach for the study of DLBCL can help in discovering novel metabolic biomarkers crucial for patients’ stratification and potentially for the development of new treatment procedures. In clinical practice, this is translated into the proximal goal of obtaining a rapid, precise, and inexpensive method for early diagnosis and prognosis prediction. To this aim, the selection of metabolites that are associated with heterogenous and not easily treatable tumors such as DLBCL will positively influence diagnosis. For the high-risk DLBCL cases with a strong MYC-driven expression signature, the selection of some key AAs influenced by MYC, such as Gln, glutamate, tyrosine, tryptophan, and BCAAs, could be initially evaluated for a general screening and later applied in routine enzymatic tests for a rapid diagnosis. The development of these metabolic “sensors” in DLBCL would be particularly important since the dissection of the molecular complexity of these cases currently requires a combination of multiple high-tech platforms, which, unfortunately, are far from being found in standardized clinical diagnostic settings. In the longer term, the use of metabolomics focused on AA metabolisms could lead to the development of new targeted treatment strategies in high-risk DLBCL as well as in other MYC-driven malignancies. Therefore, targeting AA metabolisms, trying to inhibit the transporters and enzymes that regulate the supply of AAs, could be an important therapeutic approach for clinically aggressive cancers.

## Figures and Tables

**Table 1:** Comparative overview of the advantages and disadvantages and the most recurrent metabolites detectable by analytical metabolomic techniques.

Technology	Advantages	Disadvantages	Most Metabolites Detected	Metabolites and References
**GC-MS**	High sensibility, resolution, and reproducibility Variety of commercial libraries Separation efficiency and quantitative accuracy	Requires derivatization Only suitable for small and volatile compounds	Low-molecular-weight molecules, volatile and polar such as alcohols, aldehydes ketones, lactate pyruvate amino acids, sugars, and free fatty acids	Glicyne, serine threonine glucose, aspartate, methionine, and cysteine valine pyroglutammic acid [53,54,55,56]
**LC-MS**	High sensitivity and specificity detects trace amounts of metabolites Does not require derivatization Suitable for polar and non-polar compounds Wide applicability	Ion suppression may affect accuracy Less standardization and reproducibility than GC-MS	High-molecular-weight molecules, non-volatile and less polar such as complex lipids (phospholipids sphingolipids triglycerides), peptides, proteins, and energy metabolites (NAD+/NAD)	Glutamine, glutamate [57]
**NMR** **Spectroscopy**	Non-destructive Simple sample preparation Highly reproducible Detailed structural information	Lower sensitivity than LC-MS and GC-MS Longer and more complex analysis times	Global profiling of lipids, proteins, nucleotides, and derivates (ADP, ATP, and AMP), urine metabolites, and small bioactive molecules (neurotransmitters)	Lysine, arginine [58]

## Data Availability

No new data was created.

## Abbreviations

The following abbreviations are used in this manuscript:

DLBCL	Diffuse large B-cell lymphoma
AAs	Amino acids
BCL2	B-cell lymphoma 2
BCL6	B-cell lymphoma 6
FISH	Fluorescence in situ hybridization
WHO	World Health Organization
BL	Burkitt lymphoma
IHC	Immunohistochemistry
HIF-1α	Hypoxia-inducible factor-1 alpha
BCAAs	Branched amino acids
SLC	Solute carrier
SLC1A5	Solute carrier family 1 member 5
BCAT1	Branched-chain amino acid transaminase 1
TCA	Tricarboxylic acid cycle
GEP	Gene expression profile
GCB	Germinal center B-cell
ABC	Activated B-cell
COO	Cell-of-origin
MHG	Molecular high-grade
GC	Germinal center
DHITSig	Double-hit gene expression signature
HGBL	High grade B-cell lymphoma
WES	Whole exome sequencing
CSR	Class-switch recombination
LBCLs	Large B-cell lymphomas
aSHM	Aberrant somatic hypermutation machinery
TSS	Transcription start site
Ig	Immunoglobulin
IgH	Immunoglobulin heavy chain
MCL	Mantle cell lymphoma
MBI	MYC Box I
FBXW7	F-Box and WD repeat domain containing 7
NMR	Nuclear magnetic resonance
bHLH-LZ	basic helix-loop-helix leucine zipper
CDKN2B	Cyclin dependent kinase inhibitor 2B
ARF	Alternate reading frame
MDM2	Mouse double minute 2 homolog
TP53	Tumor protein p53
TFAM	Transcription factor A
PPARGC1A	Peroxisome proliferator-activated receptor gamma coactivator 1-alpha
HK2	Hexokinase 2
PFKM	Phosphofructokinase
ENO1	Enolase 1
LDHA	Lactate dehydrogenase A
PKM2	Pyruvate kinase M2
LC-MS	Liquid chromatography–mass spectrometry
GC-MS	Gas chromatography–mass spectrometry
ND	Recently diagnosed
CR	Complete remission
α-KG	Alpha-ketoglutarate
REF	Refractory disease
REL	Early relapse
EAAs	Essential amino acids
NEAAs	Non-essential amino acids
LAT	L-type amino acid transporter
OAA	Oxaloacetate
GLS1	Glutaminase 1
GLS2	Glutaminase 2
TFEB	Transcription factor EB
Gln	Glutamine
